# Adenosquamous Carcinoma of the Gallbladder: Case Report of a Rare Malignancy

**DOI:** 10.7759/cureus.38025

**Published:** 2023-04-23

**Authors:** Michelle M Ishaya, Rafaella Litvin, Pius E Ojemolon

**Affiliations:** 1 Internal Medicine, John H. Stroger, Jr. Hospital of Cook County, Chicago, USA

**Keywords:** gallbladder adenosquamous carcinoma, malignant biliary obstruction, case report, rare malignancies, gallbladder carcinoma, adenosquamous carcinoma

## Abstract

Adenosquamous carcinoma (ASC) of the gallbladder is an incredibly rare malignancy. It is much less common than adenocarcinoma of the gallbladder and also has a much poorer prognosis. The case presented here is that of a patient diagnosed with ASC of the gallbladder after undergoing cholecystectomy for symptomatic cholelithiasis. Her disease progressed despite four cycles of chemotherapy. Her course was complicated by recurrent obstructive jaundice requiring biliary duct stent placement and percutaneous biliary drain placement over several admissions. She was discharged home with hospice service seven months after diagnosis, where she died a few weeks later. Knowledge pertaining to gallbladder ASC is limited, as prevalence is low and information is mostly derived from case reports such as this.

## Introduction

Gallbladder carcinoma (GBC) is a rare malignancy, with a prevalence of 1-2/100,000 in the United States [1]. Adenosquamous carcinoma (ASC) of the gallbladder, according to WHO classification, is a histopathological subtype comprised of both glandular and squamous components [2]. While the incidence of this subtype is not known, adenosquamous and squamous subtypes combined comprise approximately 5% of all GBCs, making ASC of the gallbladder quite rare [2]. Herein, we present the case of a patient diagnosed with ASC of the gallbladder while undergoing management for symptomatic cholelithiasis.

## Case presentation

**Figure 1:**
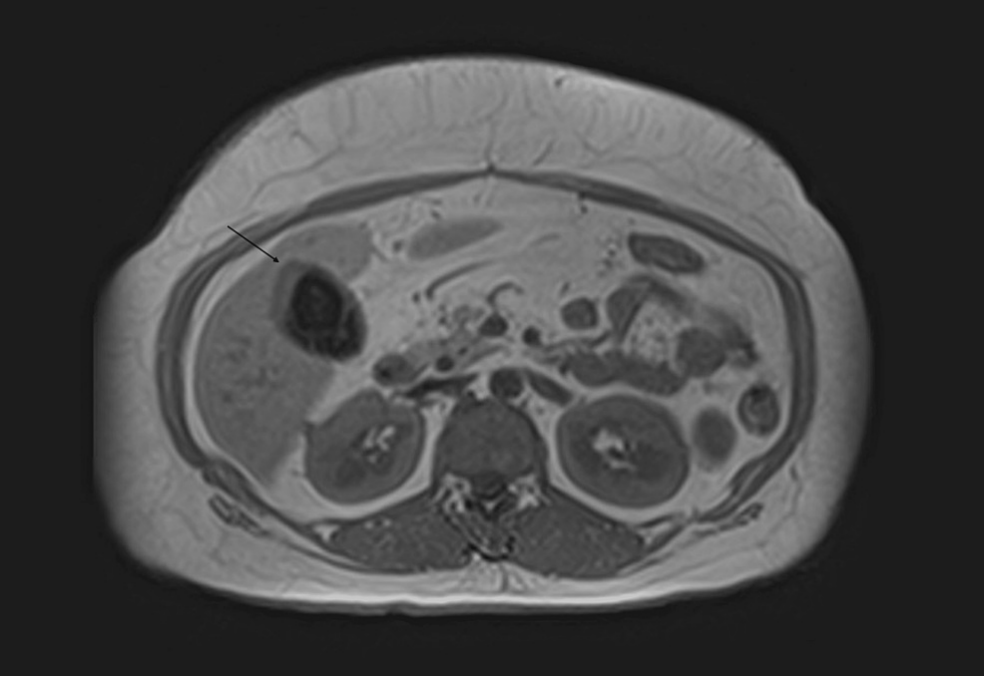
Abdominal MRI showing irregular wall thickening of the gallbladder fundus Wall thickening denoted by arrow

**Figure 2:**
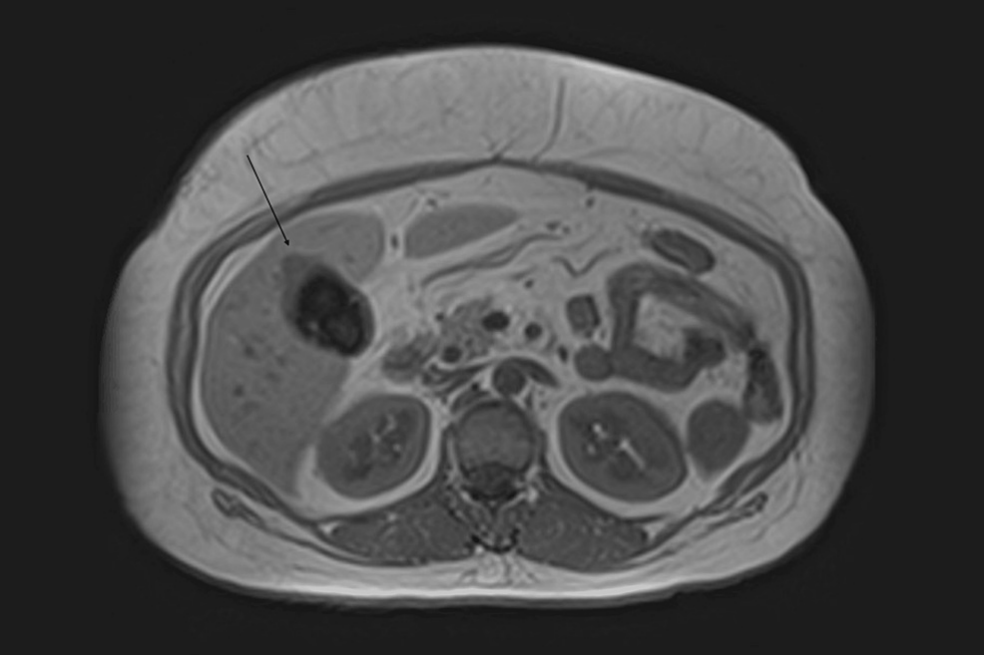
Abdominal MRI showing irregular wall thickening of the gallbladder fundus (additional image) Irregular wall thickening denoted by arrow

A 47-year-old Hispanic lady with hypertension, diabetes, dyslipidemia, cholelithiasis, and obesity presented to the emergency department with intermittent right upper quadrant and epigastric pain of three months duration. Physical examination was significant for right upper quadrant tenderness. Laboratory investigations were unremarkable, including liver profile and enzymes within normal limits. CT abdomen and pelvis with intravenous contrast showed cholelithiasis and irregular gallbladder wall thickening. MRI with cholangiopancreatography was obtained for further evaluation and showed cholelithiasis without evidence of acute cholecystitis and mild intrahepatic and extrahepatic biliary ductal dilatation without choledocholithiasis. Focal irregular wall thickening of the gallbladder fundus was also reported, measuring approximately 16 mm at the area of the maximal thickness (Figure 1-2). This was suspected to be adenomyomatosis, although malignancy could not be excluded based on imaging alone.

The patient was planned for cholecystectomy for symptomatic cholelithiasis; however, the gallbladder surface was found to have an abnormal appearance intraoperatively concerning malignancy. Gallbladder fundal wall biopsies were obtained, and frozen sections resulted positive for adenocarcinoma. Invasion of the common hepatic duct (CHD) and right and left hepatic ducts was noted, and the mass was deemed unresectable. The surgeon proceeded with palliative cholecystectomy, and the patient was admitted for additional workup, with a tentative plan to initiate chemotherapy and re-consider surgical resection pending response. CEA and CA 19-9 were initially mildly elevated and subsequently uptrended, as shown in Table 1. CA-125 was also mildly elevated on initial measurement (Table 1). After the final pathology result, the patient was diagnosed with stage IIIA ASC of the gallbladder.

**Table 1:** Tumor marker values and reference ranges *Tumor marker not measured at this time

Test	Initial value (time zero)	Value at week two	Value at week seven	Value at month six	Reference range
CEA	3.27 ng/mL	4.58 ng/mL	6.99 ng/mL	26 ng/mL	0.00-2.99 ng/mL
CA-125	N/A*	44.60 U/mL	19.6 U/mL	N/A*	0.00-35.00 U/mL
CA 19-9	64.3 U/mL	59.7 U/mL	536.9 U/mL	1012.4 U/mL	0.00-35.00 U/mL

**Figure 3:**
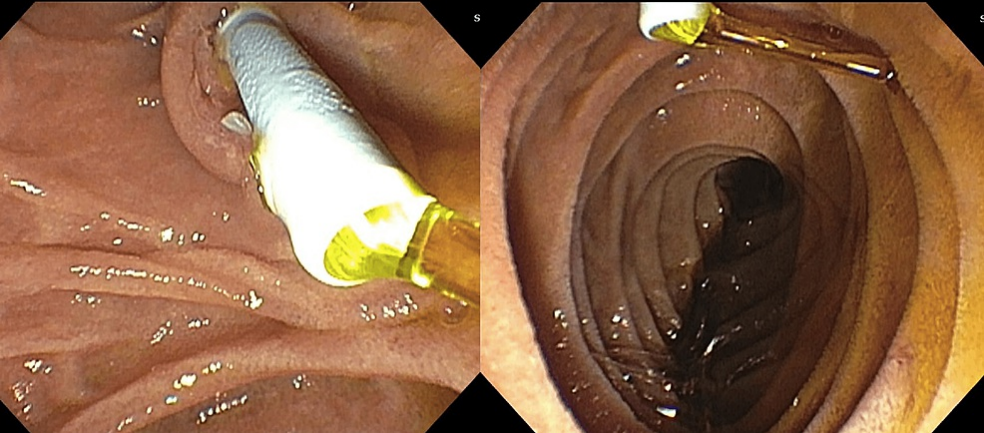
Biliary stent draining bile

Upon presentation to the oncology clinic to start chemotherapy, the patient was jaundiced and endorsed pruritus. She was admitted and found to have elevated total bilirubin, direct bilirubin, AST, ALT, alkaline phosphatase, and GGT, with values suggestive of a cholestatic pattern of injury. She was admitted and underwent endoscopic retrograde cholangiopancreatography (ERCP), revealing a high-grade stricture of the CHD which was successfully stented (Figure 3).

She received four cycles of gemcitabine and cisplatin, with durvalumab added to cycles two-four. Her course was complicated by multiple additional admissions for malignant obstructive jaundice over several months, each requiring ERCP with CHD stent exchange. Repeat CT after cycle four showed stable disease and no obvious lymphadenopathy; thus, bile duct and liver resection were planned. Unfortunately, intraoperative findings of retroperitoneal and omental metastases and portal lymphadenopathy precluded resection, and the operation was aborted.

After several ERCPs with CHD stent exchange, she underwent left-sided percutaneous biliary drain placement by interventional radiology. Despite this, the patient presented with obstructive jaundice yet again. The interventional radiology team reviewed the latest CT, which showed multi-duct biliary obstruction, and concluded that additional percutaneous drain placement would not be beneficial. Palliative care was consulted at this time, seven months after the initial presentation, and the patient was discharged home with hospice service, where she died a few weeks later.

## Discussion

Gallbladder malignancies arise from the epithelium of the gallbladder wall and can be classified as adenocarcinoma, squamous cell carcinoma (SCC), or ASC, based on histopathology. GBCs are rare, with a prevalence of 1-2/100,000 in the United States, and are more common in South America, India, Pakistan, Korea, and Japan [1,3]. Within the United States, rates are higher among Native Americans, Mexican Americans, and Whites [4]. Risk factors for the development of gallbladder cancer include female gender, obesity, diabetes, gallstones, chronic cholecystitis, PSC, certain gallbladder polyps, and porcelain gallbladder [5]. GBC often presents late and at an advanced stage and, therefore, has a poor prognosis with a five-year survival rate of less than 5% [6]. Adenocarcinoma of the gallbladder is the most common subtype, making up 90-95% of all cases of GBC [7].

Adenosquamous is a much less common histopathological subtype of GBC and is comprised of both glandular and squamous components, with the squamous component making up 25-99% of the mass [8]. ACS and pure SCC combined account for approximately 5% of GBCs [7]. It is unknown if risk factors for the development of ASC are similar to adenocarcinoma, as data are insufficient to confirm this due to the relatively small number of cases. One study, in which 34 cases of ASC and SCC were reviewed, found that the female-to-male ratio was 3.8:1, suggesting that female gender is also a factor in the development of GBC with squamous component [9]. While many believe other risk factors to be similar as well, some studies suggest that risk factors vary among different subtypes of GBC [10]. While a definitive statement cannot be made due to limitations of the current knowledge regarding ASC of the gallbladder, our patient had several diagnoses known to be risk factors for adenocarcinoma of the gallbladder. However, one area where these two groups of GBC are known to differ significantly is the prognosis.

Patients with adenosquamous and squamous have a worse prognosis than those with stage-matched advanced adenocarcinoma [9,11]. One study found a median survival time of four months in ACS/SCC vs 11.4 months in advanced-stage adenocarcinoma upon review of 606 cases of GBC, 34 of which were ACS/SCC [9]. ACS/SCC is more aggressive than gallbladder adenocarcinomas, presumably due to features of the squamous component contributing to the propensity to be more advanced at diagnosis, such as the ability to proliferate at an increased rate [1,3,12]. Adenosquamous and squamous carcinoma also demonstrates an increased rate of liver metastasis at the time of surgical resection as compared to adenocarcinoma [13]. Some studies suggest that ACS/SCC also has a higher likelihood of local lymph node involvement at the time of diagnosis [1]. Mechanisms causing ACS/SCC to be more aggressive remain unknown, but additional studies regarding differences among subtypes may help elucidate this matter in the future. For example, two pathological variations discovered in one analysis were increased bizarre pleomorphic tumor giant cells and tumor-infiltrating eosinophils in ACS/SCC as compared to adenocarcinoma cases, although the significance of this remains unclear [9].

## Conclusions

Adenosquamous carcinoma of the gallbladder is very rare, and most of what is known is derived from case reports and a few clinicopathologic reviews. Most cases are advanced at the time of diagnosis and the prognosis is poor, even more so than adenocarcinomas of the gallbladder. Much remains unknown about GBC with a squamous component, and additional information may lead to tailored therapies and the potential for more favorable outcomes for the few unfortunate patients diagnosed with adenosquamous carcinoma of the gallbladder.

## References

[1] Mandal S, Ponnekanti SK, Dadeboyina C, Tipparthi A, Kasireddy V (2021). A case report on adenosquamous carcinoma of gallbladder: a very rare malignancy. Cureus.

[2] Henson DE, Albores-Saavedra J, Code D (1992). Carcinoma of the gallbladder. Histologic types, stage of disease, grade, and survival rates. Cancer.

[3] Gulwani HV, Gupta S, Kaur S (2017). Squamous cell and adenosquamous carcinoma of gall bladder: a clinicopathological study of 8 cases isolated in 94 cancers. Indian J Surg Oncol.

[4] Rawla P, Sunkara T, Thandra KC, Barsouk A (2019). Epidemiology of gallbladder cancer. Clin Exp Hepatol.

[5] Goetze TO (2015). Gallbladder carcinoma: prognostic factors and therapeutic options. World J Gastroenterol.

[6] Pang L, Zhang Y, Wang Y, Kong J (2018). Pathogenesis of gallbladder adenomyomatosis and its relationship with early-stage gallbladder carcinoma: an overview. Braz J Med Biol Res.

[7] Shariff MH, Bhat SP, KishanPrasad HL (2016). An unusual presentation of adenosquamous carcinoma of gallbladder. Int J Pathol Lab Med.

[8] Albores-Saavedra J, Henson DE, Sobin LH (1992). The WHO histological classification of tumors of the gallbladder and extrahepatic bile ducts. A commentary on the second edition. Cancer.

[9] Roa JC, Tapia O, Cakir A (2011). Squamous cell and adenosquamous carcinomas of the gallbladder: clinicopathological analysis of 34 cases identified in 606 carcinomas. Mod Pathol.

[10] Andrea C, Francesco C (2003). Squamous-cell and non-squamous-cell carcinomas of the gallbladder have different risk factors. Lancet Oncol.

[11] Song HW, Chen C, Shen HX (2015). Squamous/adenosquamous carcinoma of the gallbladder: analysis of 34 cases and comparison of clinicopathologic features and surgical outcomes with adenocarcinoma. J Surg Oncol.

[12] Nishihara K, Takashima M, Furuta T, Haraguchi M, Tsuneyoshi M (1995). Adenosquamous carcinoma of the gall-bladder with gastric foveolar-type epithelium. Pathol Int.

[13] Chan KM, Yu MC, Lee WC, Jan YY, Chen MF (2007). Adenosquamous/squamous cell carcinoma of the gallbladder. J Surg Oncol.

